# Laparoscopic resection *vs* laparoscopic radiofrequency ablation for the treatment of small hepatocellular carcinomas: A single-center analysis

**DOI:** 10.3748/wjg.v23.i4.653

**Published:** 2017-01-28

**Authors:** Marco Casaccia, Gregorio Santori, Giuliano Bottino, Pietro Diviacco, Enzo Andorno

**Affiliations:** Marco Casaccia, Giuliano Bottino, Pietro Diviacco, Enzo Andorno, Department of Surgery, IRCCS, San Martino University Hospital-IST, University of Genoa, 16132 Genoa, Italy; Marco Casaccia, Gregorio Santori, Department of Surgical Sciences and Integrated Diagnostics, School of Medicine, University of Genoa, 16132 Genoa, Italy

**Keywords:** Hepatocellular carcinoma, Laparoscopic liver resection, Laparoscopic radiofrequency ablation, Survival, Disease-free survival

## Abstract

**AIM:**

To compare survival and recurrence after laparoscopic liver resection (LLR) and laparoscopic radiofrequency ablation (LRFA) for the treatment of small hepatocellular carcinoma (HCC).

**METHODS:**

Between June 1, 2005 and November 30, 2010, 46 patients (62.26 ± 8.55 years old; female/male: 12/34) treated for small HCC were enrolled following strict criteria. Patients with better liver function and larger tumors were referred for LLR (*n* = 24), while those with poorer liver function and multiple tumors were referred for LRFA (*n* = 22), and they were then followed for similar durations (44.74 ± 21.3 mo for LLR *vs* 40.27 ± 30.8 mo for LRFA).

**RESULTS:**

The LLR and LRFA groups were homogeneous with regard to age, sex, etiology of liver cirrhosis, and AFP levels. The overall survival (OS) and disease-free survival (DFS) probability was 0.354 and 0.260, respectively. A significantly higher OS was observed in the LLR group (LLR: 0.442; LRFA: 0.261; *P* = 0.048), whereas no statistical difference was found for DFS (LLR: 0.206; LRFA: 0.286; *P* = 0.205). In the LRFA group was treated a greater number of nodules (LLR: 1.41 ± 0.77; LRFA: 2.72 ± 1.54; *P* < 0.001). Cox regression analysis found the number of intraoperative HCC nodules as the unique variable statistically significant for OS (hazard ratio: 2.225; *P* < 0.001). The rank-hazard plot showed a steeper increase of relative hazard for intraoperative nodules > 2.

**CONCLUSION:**

Our preliminary results confirm the superiority of hepatic resection on thermoablation in the treatment of small HCC in selected patients, when both approaches are made laparoscopically. LLR showed better results compared to LRFA in terms of OS. These data need to be confirmed by further studies on a larger number of patients.

**Core tip:** This is a retrospective study to evaluate and compare the oncological results of hepatic resection and thermoablation in the treatment of small hepatocellular carcinoma in selected patients, when both approaches are made laparoscopically. Our preliminary results confirm the superiority of laparoscopic liver resection compared to laparoscopic radiofrequency ablation in terms of overall survival. These data need to be confirmed by further studies on a larger number of patients.

## INTRODUCTION

Both liver resection and ablative therapies are able to modify the natural history of hepatocellular carcinoma (HCC)[1-3]. Indeed, it is demonstrated that they allow an increase in survival in patients with small nodules, although relapse after these two treatments remains a questionable topic. However, studies in the literature comparing radiofrequency ablation to liver resection seem to confirm the superiority of the latter in terms of overall survival and disease-free survival[4-6]. To date, interstitial therapies were always compared to hepatic resection performed through an open approach. However, the fact of comparing a minimally invasive technique to a quite invasive one has some limits. Currently we are witnessing an increasing use of laparoscopy in liver surgery, extending indications to patients with cirrhosis and HCC[7,8]. Laparoscopic surgery of the liver represents a viable alternative to open surgery retaining its characteristics of a minimally invasive approach and providing better results and a similar postoperative complication rate[9,10]. Moreover, laparoscopic liver resection (LLR) in selected cirrhotic patients with HCC has shown advantages in terms of survival and recurrence similar to those after open surgery[11,12].

With the aim to compare LLR and laparoscopic radiofrequency ablation (LRFA) in terms of postoperative survival and HCC recurrence, we evaluated two groups of cirrhotic patients with similar clinical characteristics.

## MATERIALS AND METHODS

### Patient characteristics

All patients (*n =* 46) referred to our unit between June 1, 2005 and November 30, 2010 underwent an established protocol to define HCC staging. In case of severe liver impairment, the possibility of liver transplantation was considered as first option. Patient eligibility to liver transplantation did not preclude LLR or LRFA as a bridge treatment. To be included in the study patients were assessed for the severity of the liver disease following the Child-Pugh classification[13]. Plasma levels of alfa-fetoprotein (AFP) were measured. In case of presence of severe coagulation disorders or very low platelet levels (platelet count < 40 × 10^9^/L), patients were excluded from the procedure. A spiral computed tomography (CT) scan and/or gadolinium-enhanced magnetic resonance imaging (MRI) was performed in all patients to document the number, size, and segmental location of all liver lesions. Only patients with “small” HCC were taken into account. According to Yao et al[14], “small” HCC is defined by a single HCC nodule ≤ 6.5 cm, or with ≤ 3 lesions when the largest of which is ≤ 4.5 cm. The choice of LLR or LRFA was based on the tumor position: if it was located in a resectable segment, a LLR was realized; in case of deep-sited lesions requiring major hepatic resection, LRFA was indicated. Furthermore, LRFA permitted to treat lesions otherwise difficult or impossible to reach by a percutaneous approach. Each patient agreed to treatment by written consent.

### LLR group

Twenty-four HCC patients were selected to undergo LLR. These patients fell into the following conditions: (1) well compensated Child's class A/B cirrhosis; (2) esophageal varices ≤ grade 2, platelet count ≥ 40 × 10^9^/L and/or international normalized ratio > 1.5; and (3) “small” lesion accessible to the laparoscopic approach and treatable by limited resection (< 3 segments). Only one patient was operated in emergency situation for an hemoperitoneum from a ruptured HCC located in segment III. Most resections were anatomic (*i.e*., resection of 1 or more anatomic segments). Non-anatomic resections consisted of resection of less than 1 segment including the tumor and an intended 1-cm tumor-free margin. Details of the technique adopted were described elsewhere[15].

### LRFA group

Twenty-two HCC patients were selected for LRFA. These patients fulfilled at least one of the following criteria: (1) large tumours (but with a diameter < 5 cm, or multiple lesions requiring repeated punctures); (2) superficial lesions adjacent to visceral structures which could be displaced by laparoscopic maneuvers; and (3) deep-sited lesions with a very difficult or impossible percutaneous approach. Exclusion criteria were the same as for the LLR group. However, main portal branches thrombosis or a severe liver disease (Child-Pugh class C) did not contraindicated the procedure.

The surgical technique adopted for LRFA was described elsewhere[16].

The liver was investigated by intraoperative laparoscopic ultrasonography (LUS) to confirm the number and size of the lesions and define their relations with the intrahepatic vascular structures. Furthermore, a definitive histological diagnosis of both HCC and liver cirrhosis has been obtained by an intraoperative biopsy of all patients undergoing LRFA or hepatic resection.

### Patient follow-up

To assess the response to LRFA or LLR, liver US and CT scan (and/or MRI) were performed within 1 month after treatment. When no enhancement or a thin peripheral enhancement rim (representative of an inflammatory response) was observed, a complete response to LRFA was achieved. An incomplete response to LRFA was defined as persistent nodular enhancement. Post-treatment recurrence, evaluated by spiral CT scans at 3 mo and every 6 mo after treatment, was defined as the new appearance of a contrast-enhanced lesion area within 2 cm from the thermoablated nodule, or the surgical margin (local HCC recurrence), or > 2 cm from the same sites (distant HCC recurrence).

### Statistical analysis

The results were expressed as mean ± standard deviations, counts and percentages. Continuous variables were preliminarily evaluated for normal distribution with the Shapiro-Wilk test, and then compared by using the Wilcoxon-Mann-Whitney test. Categorical variables were compared with the Fisher’s exact test.

Patient overall survival (OS) and disease-free survival (DFS) were calculated with the Kaplan-Meier product-limit estimator. In the OS evaluation, the entry/final time-point corresponded to date of treatment/date of death (uncensored observations) or date of last follow-up for patients alive at the end of the study (censored observations). The final time-point for DFS was the date of the first recurrence, by censoring survival/dead patients without any recurrence. The log-rank test was applied to evaluate the differences in OS and DFS after grouping for treatments.

Cox proportional-hazard models were performed for both patient survival and DFS. In each Cox model, the Efron approximation was used for its accuracy and computational efficiency. To determine whether the fitted Cox regression models adequately described the data, the proportional-hazards assumption was tested as previously described[17,18]. Only univariate Cox regression models that did not infringe the proportional hazard assumption for OS were presented. A rank-hazard plot for the covariates of significant Cox models was created as previously described[19]. Statistical significance was assumed with a two-tailed *P* value < 0.05.

The statistical analysis was performed by using the R software/environment (version 3.3.1; R Foundation for Statistical Computing, Vienna, Austria)[20].

## RESULTS

Between June 1, 2005 and November 30, 2010, 46 patients (62.26 *±* 8.55 years old; Female/Male: 12/34) treated for small HCC were enrolled following strict criteria. Patients with similar baseline characteristics underwent LLR (*n =* 24) or LRFA (*n =* 22). Patients with better liver function and larger tumors were referred for resection, while those with poorer liver function and multiple tumors were referred for ablation. Characteristics of patients grouped for laparoscopic procedure are summarized in Table 1. The LLR and LRFA groups were homogeneous with regard to age, sex, etiology of liver cirrhosis, and AFP levels. Mean size of dominant nodule was larger and almost statistically significant in the LLR group (33 mm *vs* 26.2 mm; *P* = 0.097), whereas number of tumors was higher in the LRFA group, since 16 (72.7%) patients presented with multiple lesions in the LRFA group *vs* 4 (16.6%) patients in the LLR group (*P* < 0.001). Consequently, the mean number of treated nodules per patient was greater in the LRFA group when compared with the LLR group (2.72 ± 1.54 *vs* 1.41 ± 0.77, *P* < 0.001). Liver function impairment was more severe in the LRFA group *(P* = 0.007), where the patients classified in the Child class A were 54.5%, whereas in the LLR group they exceeded 90%. Mean follow-up duration for the LLR and LRFA groups was similar (44.74 ± 21.30 mo *vs* 40.27 ± 30.89 mo, *P* = 0.273).

**Table 1:** Patient characteristics, etiology of liver cirrhosis, Child-Pugh class, and post-operative parameters grouped for laparoscopic procedure *n* (%)

**Variables**	**LLR**	**LRFA**	***P* value**
	**(*n* = 24)**	**(*n* = 22)**	
Patient-related characteristics			
Sex (F/M)	8/16	4/18	NS
Age (yr)	63.58 ± 9.55	60.82 ± 7.25	NS
Etiology of liver cirrhosis			
HCV-related	11 (45.8)	8 (36.4)	NS
HBV-related	6 (25)	8 (36.4)	NS
Alcoholic	6 (25)	3 (13.6)	NS
Mixte	1 (4.2)	0 (0)	NS
Other	0 (0)	2 (9.1)	NS
Child-Pugh class			
Child A	22 (91.7)	12 (54.5)	0.007
Child B	2 (8.3)	6 (27.3)	NS
Child C	0 (0)	2 (9.1)	NS
Unknown	0 (0)	2 (9.1)	NS
Post-operative parameters			
Single tumour	20 (83.3)	6 (27.3)	< 0.001
Multiple tumours	4 (16.6)	16 (72.7)	< 0.001
Treated nodules/patient	1.41 ± 0.77	2.72 ± 1.54	< 0.001
Tumour diameter (mm)	33.0 ± 13.83	26.25 ± 13.13	NS
Follow-up (mo)	44.74 ± 21.30	40.27 ± 30.89	NS
Intrahepatic recurrence	16 (66.7)	15 (68.2)	NS
Local recurrence	5 (20.9)	8 (36.4)	NS
Recurrence IT (mo)	25.53 ± 19.11	22.88 ± 30.62	NS

LLR: Laparoscopic liver resection; LRFA: Laparoscopic radiofrequency ablation; HCV: Hepatitis C virus; HBV: Hepatitis B virus; IT: Interval time; NS: Not significant.

Disease recurrence was observed in a shorter time for the LLR group when compared to LRFA (25.53 ± 19.11 mo *vs* 22.88 ± 30.62 mo), even if the difference did not reach statistical significance (*P* = 0.084). In both groups, HCC recurred mainly at distance from the previous site of treatment; when considering the local recurrence rate, this was more favorable for the LLR (20.9%) than for LRFA (36.4%), although without reaching statistical significance (*P* = 0.330). Treatments for liver recurrence after LLR included chemoembolization in 9 cases, orthotopic liver transplantation in 3 cases, percutaneous radiofrequency ablation in 2 cases, a repeat resection in 2 cases (5 patients receiving 2 treatments). Recurrences after LRFA were treated with orthotopic liver transplantation in 6 cases, chemoembolization in 5 cases and liver resection in 2 cases (2 patients receiving 2 treatments).

**Figure 1:**
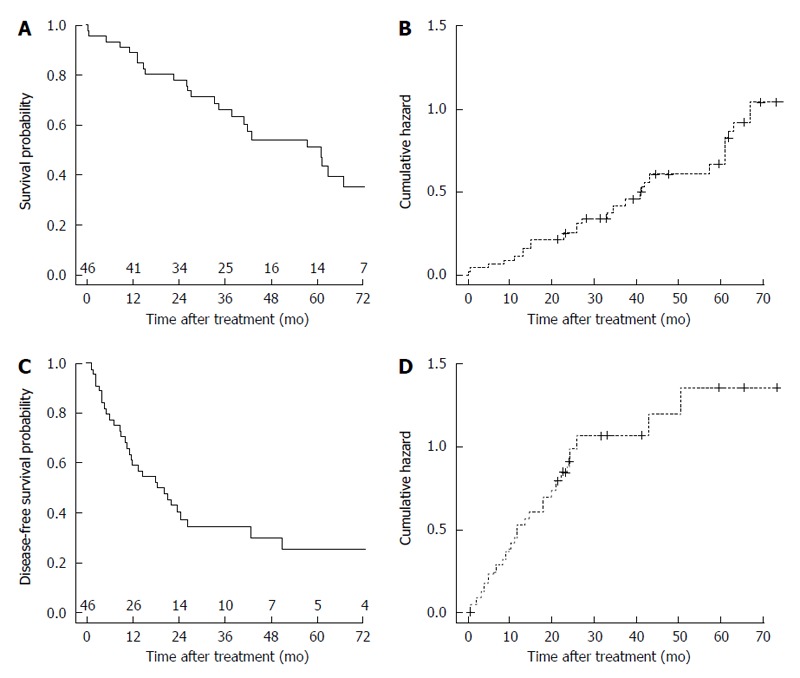
Overall and disease-free survival curves of the laparoscopically-treated hepatocellular carcinoma patients and their corresponding cumulative hazard curves. A: Overall survival curve of the laparoscopically-treated hepatocellular carcinoma (HCC) patients. Number of patients at risk at each time point is shown at top of the x-axis; B: Cumulative hazard for the corresponding overall survival curve; C: Disease-free survival curve of the laparoscopically-treated HCC patients. Number of patients at risk at each time point is shown at top of the X-axis; D: Cumulative hazard for the corresponding disease-free survival curve.

**Figure 2:**
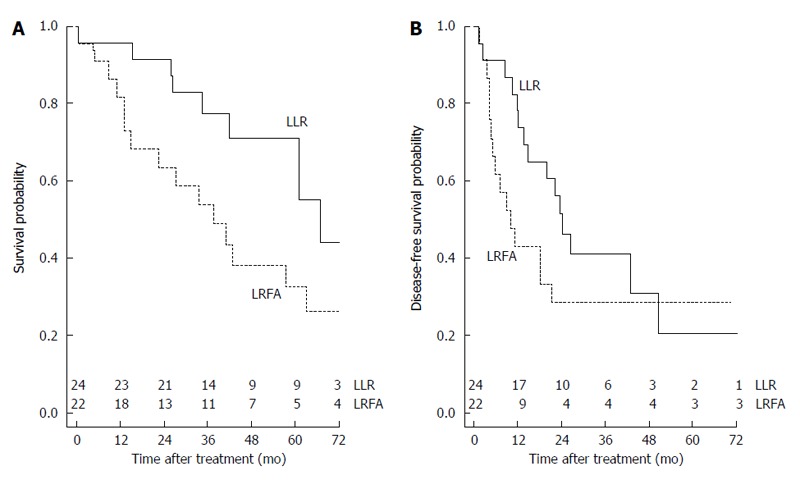
Comparison of the overall and disease-free survival probability for hepatocellular carcinoma patients by stratifying for laparoscopic treatment. A: Comparison of the overall survival probability for hepatocellular carcinoma (HCC) patients by stratifying for laparoscopic treatment (LLR: 0.442; LRFA: 0.261; *P* = 0.048). Number of patients at risk at each time point is shown at top of the x-axis; B: Comparison of the disease-free survival probability for HCC patients by stratifying for laparoscopic treatment (LLR: 0.206; LRFA: 0.286; *P* = 0.205). Number of patients at risk at each time point is shown at top of the X-axis. LLR: Laparoscopic liver resection; LRFA: Laparoscopic radiofrequency ablation.

The OS probability was 0.354 (1-year OS: 0.891; 3-year OS: 0.660; 5-year OS: 0.512) (Figure 1A). The cumulative hazard plot for the corresponding OS curve is shown in Figure 1B. The DFS probability was 0.260 (1-year DFS: 0.591; 3-year DFS: 0.346; 5-year DFS: 0.260) (Figure 1C). The cumulative hazard plot for the corresponding DFS curve is shown in Figure 1D. By stratifying for laparoscopic treatment, a significantly higher OS (*P* = 0.048) was observed in the LLR group (Figure 2A), whereas no difference for DFS (*P* = 0.205) occurred (Figure 2B).

**Figure 3:**
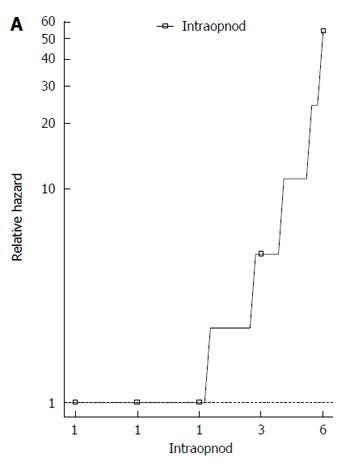
Rank-hazard plot for the number of intraoperative hepatocellular carcinoma nodules. Based on the univariate Cox model for overall survival.

The main variables were entered into univariate Cox models for OS and DFS. Only the univariate Cox regression models that did not infringe the proportional hazard assumption for OS were reported in Table 2. The number of intraoperative HCC nodules was the unique variable statistically significant for OS (HR = 2.225; *P* < 0.001). The corresponding rank-hazard plot showed a steeper increase of relative hazard for intraoperative nodules > 2 (Figure 3). Univariate Cox analysis for DFS did not find any significant predictor (Table 2).

**Table 2:** Cox proportional hazard regression for overall survival and disease-free survival

**Variables**	**β**	**HR (95%CI)**	***P* value**
Overall survival			
Treatment (LRFA)	0.812	2.252 (0.983-5.156)	NS
Sex (Male)	0.073	1.076 (0.426-2.717)	NS
Age	0.008	1.008 (0.961-1.059)	NS
Diagnosis (HCV)	0.828	2.288 (0.624-8.382)	NS
Preop TACE (Yes)	0.500	1.649 (0.737-3.689)	NS
No. intraop. nodules	0.800	2.225 (1.594-3.108)	< 0.001
Local recurrence (Yes)	-0.014	0.985 (0.414-2.345)	NS
Dist. recurrence (Yes)	1.543	4.680 (0.618-35.440)	NS
Disease-free survival			
Treatment (LRFA)	0.464	1.590 (0.771-3.282)	NS
Sex (Male)	0.587	1.798 (0.732-4.422)	NS
Age	-0.019	0.980 (0.939-1.024)	NS
Diagnosis (HCV)	-0.300	0.740 (0.275-1.993)	NS
Preop TACE (Yes)	0.125	1.133 (0.549-2.340)	NS
No. intraop. nodules	0.216	1.241 (0.946-1.628)	NS

The square brackets show the target code selected for each categorical variable. β: Regression coefficient; LRFA: Laparoscopic radiofrequency ablation; HCV: Hepatitis C virus; TACE: Transarterial chemoembolization; NS: Not significant.

## DISCUSSION

There is some dispute whether survival benefits of RFA compared with liver resection exist for patients with HCC conforming to the Milan criteria. Prospective randomized trials comparing these two procedures for HCC conforming to the Milan criteria showed controversial results[4,21,22]. A recent meta-analysis by Yi et al[23] showed that liver resection was superior to RFA in the treatment of patients with HCC conforming to the Milan criteria in terms of 3 and 5-year survival rates and local recurrence rate, suggesting that liver resection remains the better choice of treatment for small HCC. The laparoscopic approach to RFA has proved to be superior to the percutaneous approach in lesions that are difficult or impossible to be treated in such a way or in severe liver disease, thus extending its indications. Despite good results, LRFA yelded inferior OS and DFS rates when compared to hepatic resection for small HCC[2,24,25].

In the lights of comparable oncological results following liver resection regardless the type of surgical approach, laparoscopic or “open”[12], we wanted to verify that the superiority of liver resection on thermoablation in terms of OS and DFS persisted even when both approaches were laparoscopic. In our study, different indications to LRFA and LLR were responsible for the slightly different patients characteristics of the two groups. In fact, patients with normal liver function and larger tumors were resected, whereas those with liver dysfunction, multiple tumors, and portal hypertension were ablated. Another limitation are the various therapies used to treat tumor recurrences in both LRFA and LLR groups, including repeat RFA, transarterial chemoembolization (TACE), second resection, liver transplantation, systemic chemotherapy, and supportive treatment. Different therapeutic schedules for tumor recurrences could affect these findings. High rate of intrahepatic recurrence after ablation therapies and/or surgical resection is the main cause of late death of patients with HCC. In current study, local recurrence was found to be more frequent after LRFA than LLR. Local recurrences after LRFA may be attributable to insufficient ablation of the primary tumor and/or the presence of tumor venous invasion in the adjacent liver. Surgical resection could remove the primary tumor and venous tumor thrombi. This may explain the better outcomes following LLR. A significantly higher OS was observed in the LLR group in comparison with LRFA group. Our results are comparable to literature data[21,26], reflecting the superiority of the resection over thermoablation, thus confirming that this relation is maintained also if a laparoscopic approach is used. Similarly, better results in 1- and 3-year DFS were observed in the LLR group in comparison with LRFA group, but this difference disappeared after 5 years.

In literature, factors that are reported to be responsible for influencing the OS and DFS are tumor size, number of tumors, Child class, AFP levels and treatment modality[21-23]. Besides the underlying liver function, the most important parameter affecting the overall survival is the number of tumor nodules, as testified by many studies comparing open liver resection to thermoablation[21,22,27]. This statement is also confirmed in our study. In fact, Cox regression analysis demonstrated that the only independent risk factor associated with OS was the intraoperative number of HCC nodules. Moreover, the corresponding rank-hazard plot showed a steeper increase of relative hazard for intraoperative nodules > 2, confirming the finding of Liu et al[27]. Thanks to these results, many centers are adopting a more aggressive approach to resection when possible as opposed to RFA[28,29]. Laparoscopic liver resection, if feasible, provides many of the advantages of a less invasive procedure with complete tumor extirpation and assessment of resection margins, thus preserving better oncological results over thermoablation.

Our preliminary results suggest that LLR for HCC is feasible with good oncologic results. Laparoscopy should be routinely considered in selected patients in centers experienced in liver surgery and in advanced laparoscopy. Better survival of hepatic resection on thermoablation in the treatment of small HCC are confirmed also when both approaches are made laparoscopically. These data need to be confirmed by further studies on a larger number of patients.

## COMMENTS

### Background

Both liver resection and ablative therapies are able to modify the natural history of hepatocellular carcinoma (HCC). Indeed, it is demonstrated that they allow an increase in survival in patients with small nodules, although relapse after these two treatments remains a questionable topic. However, studies in the literature comparing radiofrequency ablation to liver resection seem to confirm the superiority of the latter in terms of overall survival and disease-free survival. To date, interstitial therapies were always compared to hepatic resection performed through an open approach. However, the fact of comparing a minimally invasive technique to a quite invasive one has some limits. Currently the authors are witnessing an increasing use of laparoscopy in liver surgery, extending indications to patients with cirrhosis and HCC. Laparoscopic surgery of the liver represents a viable alternative to open surgery retaining its characteristics of a minimally invasive approach and providing better results and a similar postoperative complication rate. Moreover, laparosopic liver resection in selected cirrhotic patients with HCC, has shown advantages in terms of survival and recurrence similar to those after open surgery. With the aim to compare laparoscopic liver resection (LLR) and laparoscopic radiofrequency ablation (LRFA) in terms of postoperative survival and HCC recurrence, we evaluated two groups of cirrhotic patients with similar clinical characteristics.

### Research frontiers

In the lights of comparable oncological results following liver resection regardless the type of surgical approach, laparoscopic or “open”, the authors wanted to verify that the superiority of liver resection on thermoablation in terms of overall survival (OS) and disease-free survival (DFS) persisted even when both approaches were laparoscopic.

### Innovations and breakthroughs

In this study, better outcomes are obtained following LLR. A significantly higher OS was observed in the LLR group in comparison with LRFA group. These results are comparable to literature data, reflecting the superiority of the resection over thermoablation, thus confirming that this relation is maintained also if a laparoscopic approach is used.

### Applications

Current preliminary results suggest that LLR for HCC is feasible with good oncologic results. Laparoscopy should be routinely considered in selected patients in centers experienced in liver surgery and in advanced laparoscopy. Better survival of hepatic resection on thermoablation in the treatment of small HCC are confirmed also when both approaches are made laparoscopically. These data need to be confirmed by further studies on a larger number of patients.

### Peer-review

It’s a good study, with a good design and a robust results however the groups in relation to lesions (number and localization) were different that results, maybe, in different survival.
